# PFKFB3-mediated glycolysis rescues myopathic outcomes in the ischemic limb

**DOI:** 10.1172/jci.insight.139628

**Published:** 2020-09-17

**Authors:** Terence E. Ryan, Cameron A. Schmidt, Michael D. Tarpey, Adam J. Amorese, Dean J. Yamaguchi, Emma J. Goldberg, Melissa M.R. Iñigo, Reema Karnekar, Allison O’Rourke, James M. Ervasti, Patricia Brophy, Thomas D. Green, P. Darrell Neufer, Kelsey Fisher-Wellman, Espen E. Spangenburg, Joseph M. McClung

**Affiliations:** 1 East Carolina Diabetes and Obesity Institute,; 2 Department of Physiology,; 3 Department of Cardiovascular Science, and; 4 Division of Surgery, East Carolina University, Brody School of Medicine, Greenville, North Carolina, USA.; 5 Department of Biochemistry, Molecular Biology and Biophysics, College of Biological Sciences, University of Minnesota, Saint Paul, Minnesota, USA.

**Keywords:** Muscle Biology, Vascular Biology, Atherosclerosis, Cardiovascular disease, Glucose metabolism

## Abstract

Compromised muscle mitochondrial metabolism is a hallmark of peripheral arterial disease, especially in patients with the most severe clinical manifestation — critical limb ischemia (CLI). We asked whether inflexibility in metabolism is critical for the development of myopathy in ischemic limb muscles. Using Polg mtDNA mutator (D257A) mice, we reveal remarkable protection from hind limb ischemia (HLI) due to a unique and beneficial adaptive enhancement of glycolytic metabolism and elevated ischemic muscle PFKFB3. Similar to the relationship between mitochondria from CLI and claudicating patient muscles, BALB/c muscle mitochondria are uniquely dysfunctional after HLI onset as compared with the C57BL/6 (BL6) parental strain. AAV-mediated overexpression of PFKFB3 in BALB/c limb muscles improved muscle contractile function and limb blood flow following HLI. Enrichment analysis of RNA sequencing data on muscle from CLI patients revealed a unique deficit in the glucose metabolism Reactome. Muscles from these patients express lower PFKFB3 protein, and their muscle progenitor cells possess decreased glycolytic flux capacity in vitro. Here, we show supplementary glycolytic flux as sufficient to protect against ischemic myopathy in instances where reduced blood flow–related mitochondrial function is compromised preclinically. Additionally, our data reveal reduced glycolytic flux as a common characteristic of the failing CLI patient limb skeletal muscle.

## Abstract 2

Supplementary glycolytic flux is sufficient to protect against ischemic myopathy when reduced blood flow-related mitochondrial function is compromised pre-clinically.

## Introduction

Peripheral arterial disease (PAD) is the third leading cause of atherosclerotic cardiovascular mortality (1) and a disease whose global incidence has increased substantially in the last 20 years (2). Despite a marked increase in the number of lower-extremity revascularization procedures, there has been no corresponding change in morbidity or mortality in these patients. Identifying the cellular processes that either contribute to or protect against ischemic injury are key to understanding the pathologic burden of CLI.

The ability to generate and maintain a cellular energy charge (ATP/ADP) is a major challenge faced by ischemic cells. During times of reduced oxygen tension, cells must be highly efficient in their utilization of existing oxygen for aerobic ATP production and/or possess the flexibility to switch to nonaerobic metabolism to maintain cellular energy charge. Metabolic inflexibility culminates in necrotic cell death caused by a decline in cellular ATP, leading to insufficient ATP-dependent ion pump activity within the cell membrane and swelling of the cell. On this basis, the clinical importance of metabolic health is highlighted by the fact that necrotic lesions and nonhealing ulcers are the primary causes of limb amputation in critical limb ischemia (CLI) patients. Published (3–7) data suggest that mitochondrial-derived disruptions in skeletal muscle energy charge are factors in the etiology of, and susceptibility to, preclinical and clinical ischemic myopathy. To this end, targeting cell metabolism in the context of PAD is a logical avenue of therapeutic discovery for limb salvage. This is even more pertinent, given the propensity of PAD patients to be aged, suffer from comorbidities (diabetes), make unhealthy lifestyle decisions (smoking, sedentary behavior), and be prescribed medications for other health problems (statins, metformin) with known independent effects on skeletal muscle metabolism (8, 9).

In this study, we set out to investigate a causal role for fragile mitochondrial function on the severity of perfusion deficits, muscle necrosis, and limb muscle myopathy following a murine model of hind limb ischemia (HLI). We simply asked whether compromised mitochondrial function would alter the survival and recovery of ischemic limb muscle. To answer this question, we first employed a transgenic line of mice harboring a mutation in the mitochondrial polymerase γ (D257A; mtDNA mutator) that results in accumulated mitochondrial DNA mutations and compromised oxygen consumption (10, 11). We found that aged, homozygous mtDNA mutator mice are capable of reprograming cellular metabolism toward a greater reliance on glycolysis, resulting in remarkable protection from ischemic myopathy. RNA sequencing (RNA-seq) and metabolic phenotypings identified expression levels of 6-phosphofructo-2-kinase/fructose-2,6-bisphosphatase 3 (PFKFB3) as a potential mediator of this protection. We next explored whether enhanced glycolytic capacity could provide a therapeutic angle for the prevention of myopathy and the beneficial restoration of limb blood flow after HLI. These additional studies confirmed a critical role for PFKFB3 in mediating metabolic flexibility and, ultimately, limb myopathy after HLI. Investigation of muscle samples from CLI patients who possess significant deficits in mitochondrial function (5) revealed a secondary unique deficit in glucose metabolism. These data demonstrate that downregulations across the glycolytic pathway compound ischemic mitochondriopathy, providing a potential dividing line between the severity of myopathic presentation in the ischemic limb. Together, these findings point to glycolytic flux as a potential therapeutic avenue in instances of ischemic muscle mitochondrial dysfunction.

## Results

### D257A^+/+^ mice have enhanced recovery of limb perfusion after HLI and are resistant to ischemic muscle myopathy.

**Figure 1:**
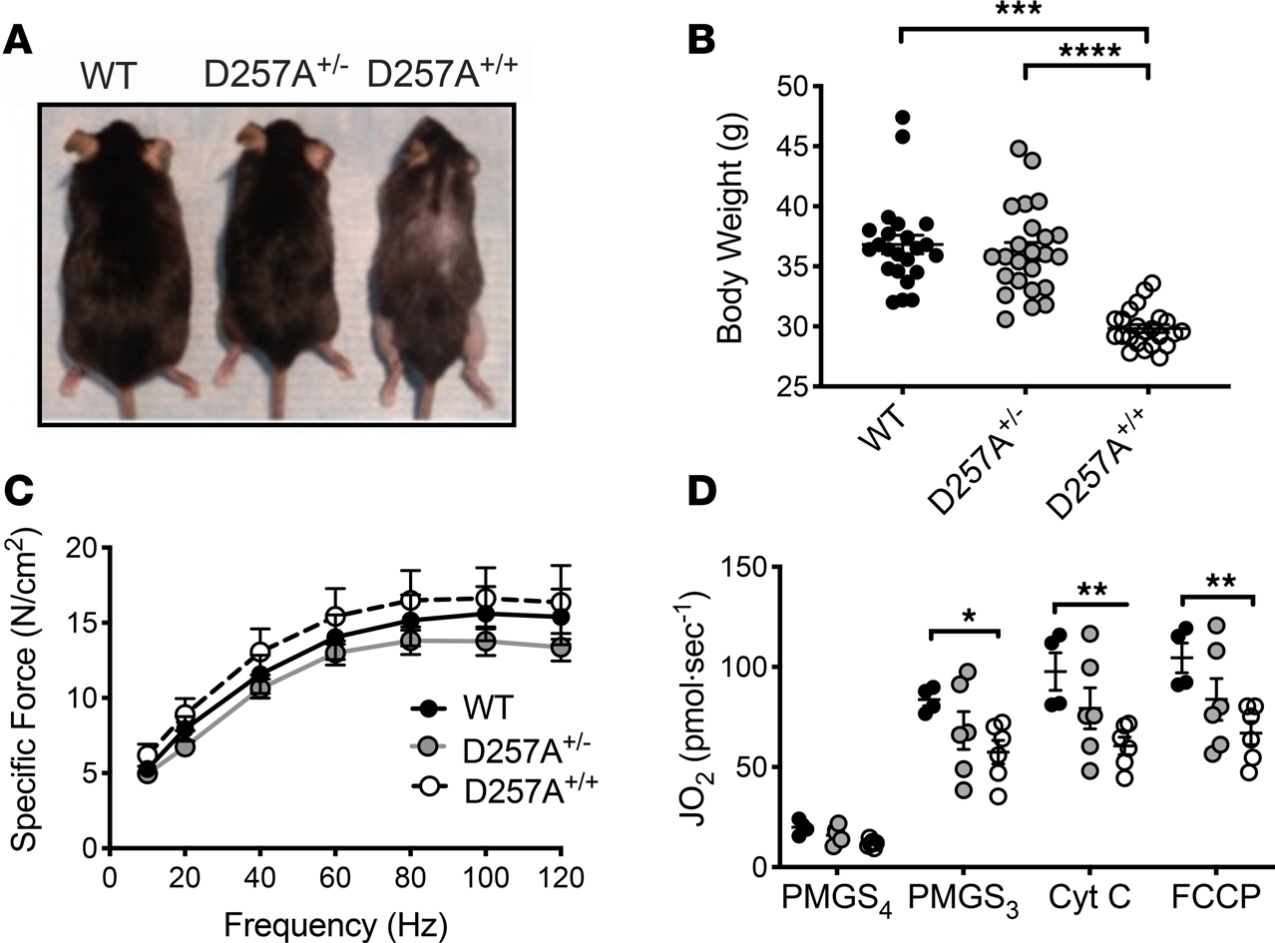
D257A^+/+^ (mtDNA mutator) mice display a distinct phenotype. WT, heterozygous (D257A^+/–^), and homozygous (D257A^+/+^) mice were aged to 12 months. (**A**) Visual representation of aged mice demonstrates typical phenotype (graying of hair, weight loss, kyphosis). (**B**) Quantification of body weight at 12-months of age. (**C**) Quantification of skeletal muscle contractile function in cage-control transgenic mice. (**D**) Mitochondrial phenotyping in mtDNA mutator mice. Mitochondria were isolated from hind limb skeletal muscle of control limbs for the direct analysis of respiratory capacity. *P < 0.05, **P < 0.01, ***P < 0.001, and ****P < 0.0001 using ANOVA (1-way in **B** and **C**, 2-way in **D**) with Tukey’s post hoc for comparisons. Values are presented as mean ± SEM.

A transgenic mouse harboring a mutant allele of mtDNA polymerase γ (D257A, The Jackson Laboratory, stock no. 017341) was subjected to HLI, facilitating the examination of the effects of progressive lifelong mitochondrial dysfunction on ischemic myopathy. Homozygous D257A mice (D257A^+/+^) experience a gradual accumulation of mtDNA mutations that reduces mitochondrial function and shortens the life span to ~13–15 months (11). D257A^+/+^, D257A^+/–^, and WT (D257A^–/–^) littermates were aged to 12 months before HLI (3, 12). Animals were carefully observed before HLI to validate previously reported phenotypes (weight loss, graying and loss of fur, kyphosis) after genotyping during the aging process (Figure 1, A and B). Quantification of skeletal muscle contractile function in cage-control transgenic mice revealed no significant reductions in isometric EDL specific force (Figure 1C). Isolated mitochondria from the control limbs further confirmed the expected mutation-induced reduction in mitochondrial function in D257A^+/+^ mice (Figure 1D).

**Figure 2:**
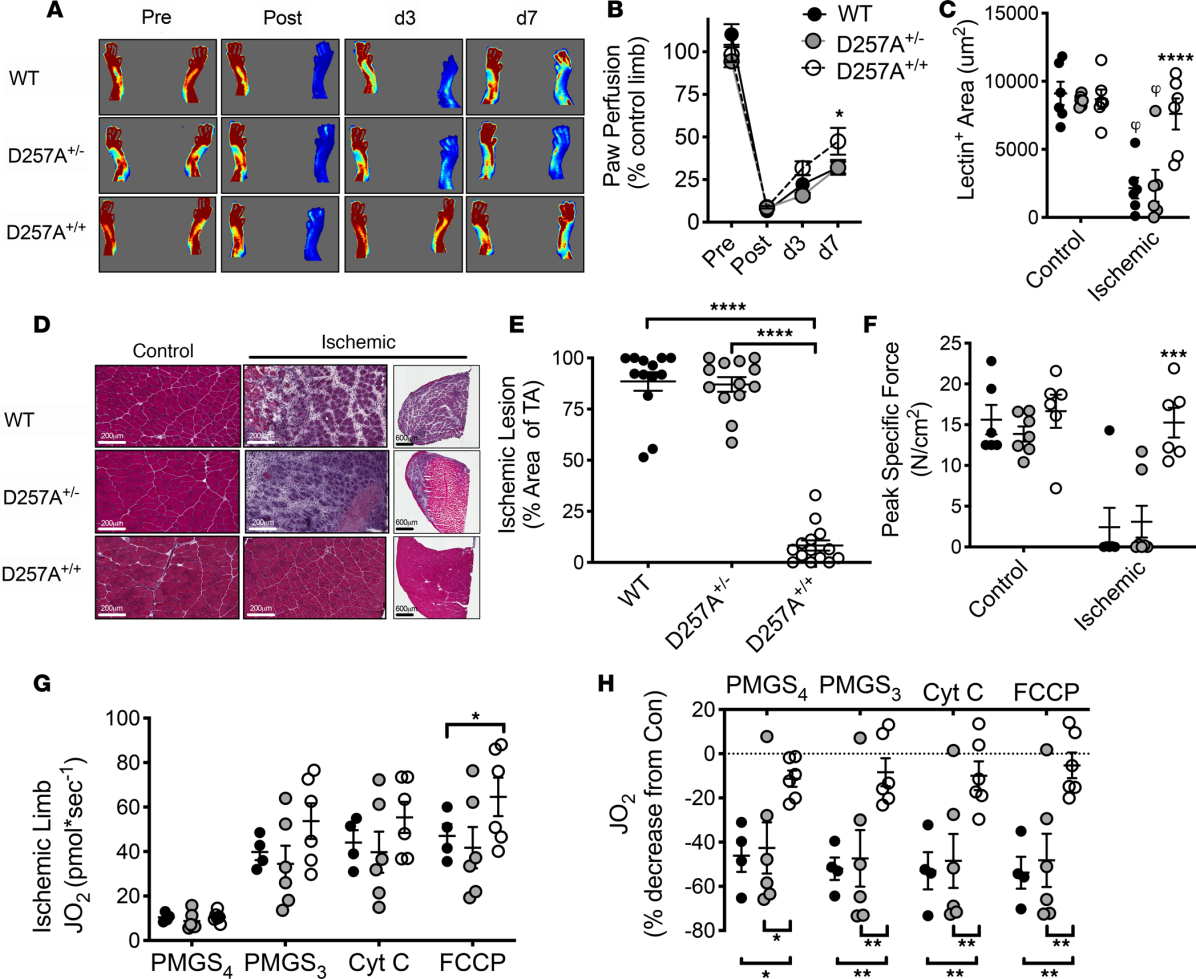
D257A^+/+^ (mtDNA mutator) mice are protected from ischemic muscle injury. Mice were aged to 12 months before performing unilateral hind limb ischemic (HLI). (**A**) Following HLI, homozygous mutants displayed greater limb perfusion recovery (**A** and **B**, *n* = 14/group) and increased perfused capillary area (**C**, *n* = 6/group; HLI d7), protection against ischemic injury to the skeletal muscle (**D** and **E**, *n* = 13 for WT and D257A^+/–^, *n* = 14 for D257A^+/+^), and no impairment of skeletal muscle contractile function in ischemic EDL muscles (**F**, *n* = 6–7/group; HLI d7). (**G**) Respiratory function in ischemic limb muscle mitochondria supported by pyruvate, malate, glutamate, and succinate under state 4 (S4) and S3 (ADP-stimulated) conditions. (**H**) Percent change in mitochondrial respiration (from control limb). *P < 0.05, ***P < 0.001, ****P < 0.0001 as indicated or versus WT (between group) using ANOVA (1-way in **E**, 2-way in **B**, **C**, **F**, **G**, and **H**) with Tukey’s post hoc for comparisons. ^φ^*P* < 0.05 versus nonischemic control (within group) using 2-way ANOVA with Tukey’s post hoc for comparisons. Values are presented as mean ± SEM.

It should be noted that the C57BL/6J strain (BL6) used as breeding mates for the D257A strain, as compared with the BALB/c parental strain, experiences minimal tissue loss and a linear recovery of limb blood flow after 7 days of HLI (12–17) when these mice are used as adults (10–15 weeks of age). Limb necrosis was minimal and largely uniform among D257A^+/+^, D257A^+/–^, and WT littermates after HLI. Importantly, the degree of ischemia induced by HLI, confirmed by laser Doppler perfusion imaging (LDPI) immediately following surgery, was similar across all groups. Surprisingly, we observed a greater recovery of paw blood flow/LDPI signal in D257A^+/+^ mice compared with either D257A^+/–^ or WT littermates (Figure 2, A and B). This finding was paralleled by the maintenance of perfused capillary density within the tibialis anterior (TA) muscle at 7 days after HLI (Figure 2C) in D257A^+/+^ homozygous mice. D257A^+/+^ mice were also completely protected from ischemic myopathy, as demonstrated by marked reductions in the ischemic lesion areas (88.5% ± 4.5% versus 8.2% ± 2.5% area of the TA muscle for WT and D257A^+/+^ mice, respectively; Figure 2, D and E) and normal muscle contractile function (Figure 2F) after 7 days of HLI. This is a stark contrast to the myopathy observed in tissues from the aged WT or D257A^+/–^ mice at this time point of HLI. HLI-induced mitochondrial impairments were significantly lower in D257A^+/+^ mice compared with littermates (Figure 2, G and H), potentially due in part to the lower mitochondrial respiratory capacity at baseline due to the transgene.

Overall, our initial experiments revealed that aged D257A^+/+^ mice exhibit a unique protective phenotype in response to HLI, despite reduced mitochondrial respiratory capacity. In comparison with their WT and D257A^+/–^ littermates, D257A^+/+^ mice are characterized by an accelerated recovery of limb blood flow, a protection of limb muscle capillary perfusion, reduced myopathic lesions, and muscle contractile functional capabilities similar to nonischemic values. Interestingly, all of these characteristics occurred despite persistently reduced (>60%) limb blood flow (compared with the contralateral control limb) across all groups. These results, while unexpected, revealed D257A^+/+^ mice maintain a clinical presentation akin to nonsymptomatic occlusive patients. This presentation is defined by sustained tissue perfusion and functional capacity representative of a resistance to ischemic myopathic injury.

### D257A^+/+^ mice adapt to progressive mitochondrial dysfunction by enhancing glycolytic metabolism.

**Figure 3:**
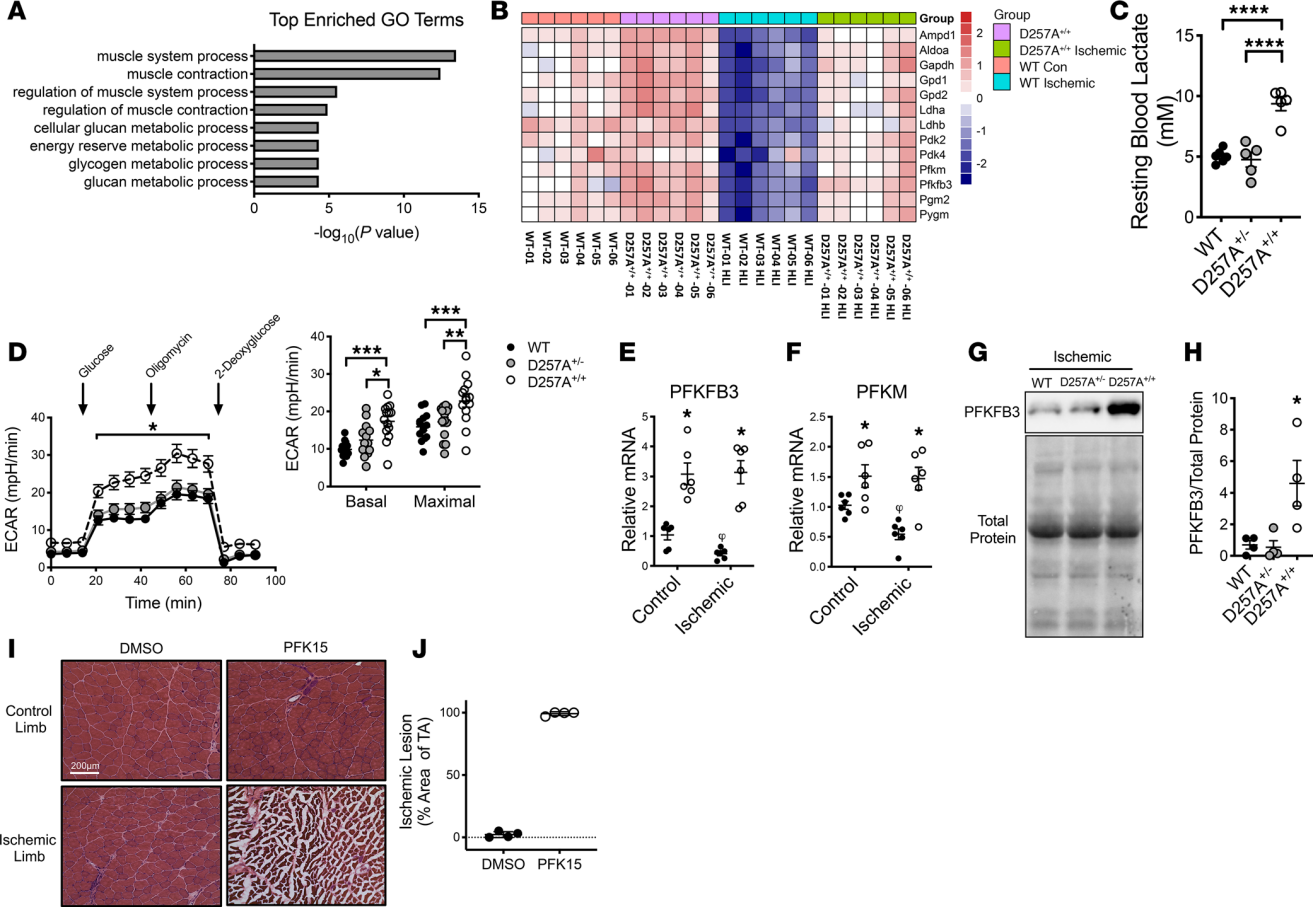
D257A^+/+^ (mtDNA mutator) mice are metabolically reprogrammed to enhance limb muscle glycolysis. (**A**) Top enriched gene ontology (GO) terms obtained from differentially expressed genes suggest a reprogramming of glycolytic metabolism. (**B**) Heatmap of top glycolytic genes. (**C–F**) Values are reported as the log_2_ of the fold change from the WT control limb. D257A^+/+^ also had increased blood lactate (**C**, *n* = 5–6/group), elevated glycolytic flux over time in primary muscle cells (**D**, *n* = 14/group; graph to the right shows basal and maximal rates), increased mRNA expression of PFKFB3 (**E**, *n* = 6/group) and PFKM (**F**, *n* = 6/group), as well as increased PFKFB3 protein expression (**G** and **H** *n* = 4/group). *P < 0.05, ** *P* < 0.01, *** *P* < 0.001, and ****P < 0.0001 versus WT using ANOVA (1-way in **C** and **H**, 2-way in **D–F**) with Tukey’s post hoc for comparisons. ^φ^*P* < 0.05 versus nonischemic control using 2-way ANOVA with Tukey’s post hoc for comparisons. Values are presented as mean ± SEM. Homozygous D257A^+/+^ mice were given 25 mg/kg PFK15, a small molecule specific inhibitor of PFKFB3, via i.p. injection 24 hours before HLI to inhibit PFKFB3, or equal volume DMSO/saline mixture as control. (**I** and **J**) PFKFB3 inhibition resulted in a substantial increase in ischemic muscle necrosis but, importantly, did not alter muscle histomorphology in the nonischemic control limb. Scale bar: 200 μm. ****P < 0.0001 versus DMSO. *n* = 4/group using 2-tailed Student’s *t* test. Values are presented as mean ± SEM.

To identify potential mechanisms underlying the unique ischemic presentation observed in the homozygous mice, whole transcriptome (mRNA) sequencing and differential gene expression analysis was performed in control and ischemic limb muscles (3 days after surgery) from both WT and D257A^+/+^ mice. The Top Enriched Gene Ontology (GO) terms were related to either skeletal muscle structure/function or glucan (polysaccharides of glucose) metabolism (Figure 3A). Gene expression related to skeletal muscle structure/function was uniformly downregulated in the ischemic muscle of WT mice (consistent with the degree of ischemic injury). D257A^+/+^ mice displayed elevated expression of several genes involved in nonoxidative metabolism in their control limbs and preservation of this expression following HLI (Figure 3B). Given this finding, we next performed metabolic phenotyping and observed a robust elevation in D257A^+/+^ resting blood lactate levels (Figure 3C), enhanced glycolytic flux (extracellular acidification rate [ECAR] measured using a Seahorse XF analyzer and corrected for nonglycolytic rates in presence of 2-deoxyglucose) in isolated D257A^+/+^ primary skeletal muscle cells (Figure 3D), increased PFKFB3 and PFKM mRNA (Figure 3, E and F) in D257A^+/+^ limb muscles at baseline and after HLI, and increased PFKFB3 protein in ischemic D257A^+/+^ muscle (Figure 3, G and H).

Because PFKFB3 is a well-known glycolytic activator (18), we next asked whether PFKFB3 (and glycolysis) was required for the ischemic protection that occurs in these mice. Aged (12-month-old) D257A^+/+^ mice were treated with PFK15, a small molecule specific inhibitor of PFKFB3 (19). Prolonged (>4 days) exposure to PFK15 proved to be fatal in D257A^+/+^ mice, suggesting that PFKFB3-mediated metabolism is crucial for their survival. Mice were monitored twice daily following HLI. PFK15 (25 mg/kg) or vehicle control (DMSO/saline mixture) were delivered by i.p. injection 24 hours before HLI and again 2 days after HLI. PFK15 treatment alone did not alter muscle histomorphology in the nonischemic control limb. Ischemic muscle injury and necrosis were significantly increased (Figure 3, I and J), indicating a necessity for PFKFB3 in conferring the ischemic myopathic resistance to D257A^+/+^ mouse limb muscles.

### Rapid-onset and chronic skeletal muscle mitochondrial dysfunction in ischemic BALB/c mice.

**Figure 4:**
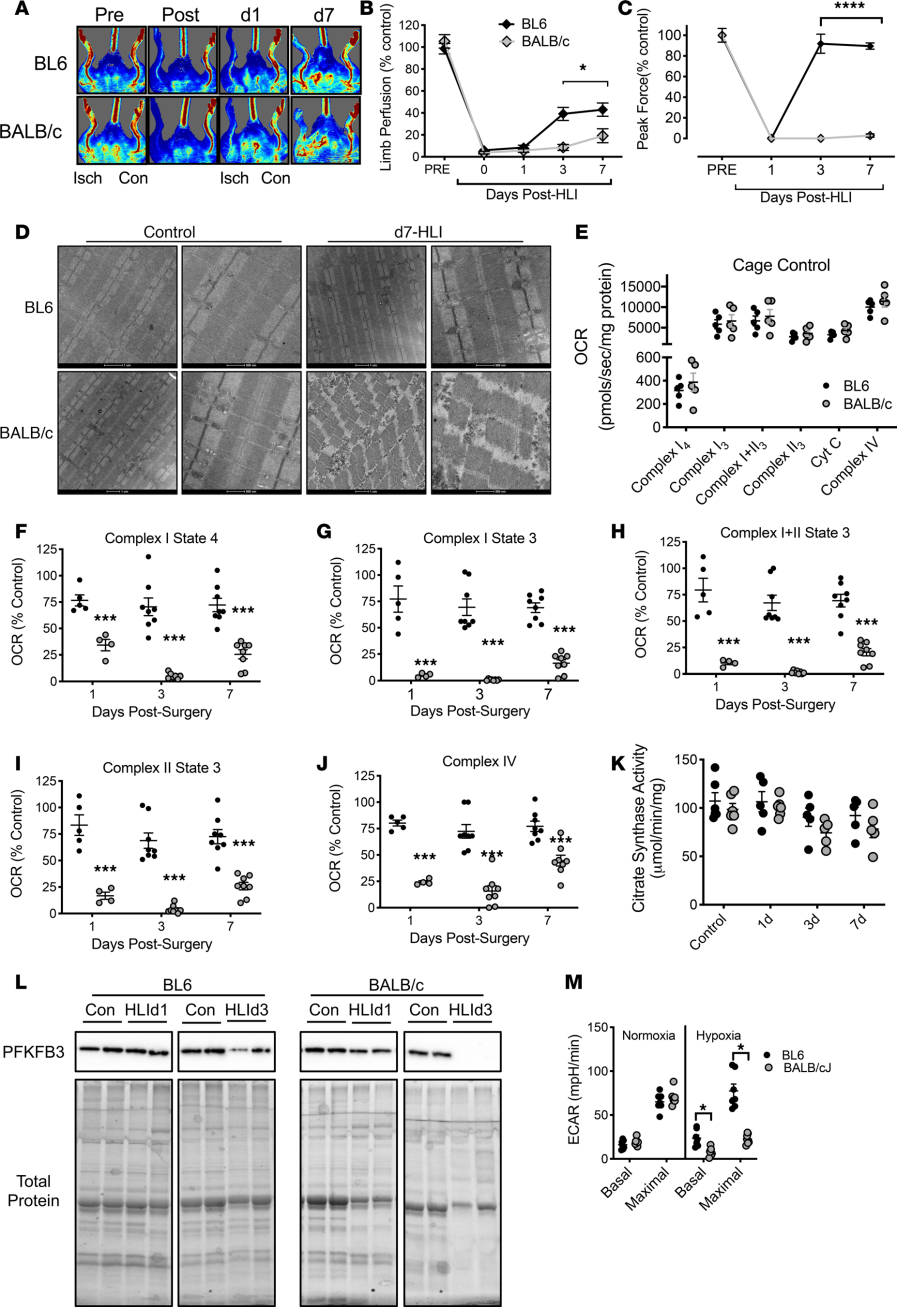
Chronic mitochondrial dysfunction in skeletal muscle of BALB/c mice. (**A**) Representative laser Doppler perfusion images (LDPI) from BL6 and BALB/c mice prior to and during the recovery from unilateral hind limb ischemia surgery (HLI). (**B**) Quantification of LDPI limb perfusion. (**C**) Ex vivo force production (expressed as a percentage of the contralateral control limb) measured in the extensor digitorum longus muscle of BL6 and BALB/c. Mitochondrial function was assessed using high-resolution respirometry in mitochondria isolated from the plantarflexor muscles. (**D**) Representative electron micrographs of the tibialis anterior muscle of BL6 and BALB/c mice under control and ischemic (HLI day 7) conditions. Scale bars: 1 µm of 500 nm. (**E**) Mitochondrial respiratory function was not different between BL6 and BALB/c mice under nonsurgical conditions. (**F**) Oxygen consumption rate (OCR) in the presence of 10 mM glutatmate + 0.5 mM malate. (**G**) Complex I supported state 3 respiration in the presence of 10 mM glutatmate + 0.5 mM malate + 4mM ADP. (**H**) State 3 respiration supported by 10 mM glutatmate + 0.5 mM malate + 10 mM Succinate + 4mM ADP. (**I**) Complex II supported state 3 respiration was assessed by inhibiting complex I with 10 μM rotenone. (**J**) Complex IV supported respiration was assessed in the presence of 2 mM ascorbic acid + 0.4mM N, N, N’, N’- tetramethyl-p-phenylenedamine (TMPD). (**K**) Citrate synthase activity in isolated mitochondria did not change with HLI. OCR is expressed as a percentage of the normoxic/normal growth media control OCR for each cell type. (**L**) Western blotting was performed on BL6 and BALB/c muscle lysates after HLI (*n* = 4, each strain). Western blot images for PFKFB3 and total protein on PVDF membrane. (**M**) Glycolytic flux (ECAR, extracellular acidification rate using Seahorse XF) in primary myotubes under normoxic and hypoxic conditions (3-hour treatment). *P < 0.05, ***P < 0.001, and ****P < 0.0001 using 2-way ANOVA with Tukey’s post hoc for comparisons. Values are presented as mean ± SEM.

In order to effectively identify the therapeutic potential of metabolic flexibility–related enhanced glycolytic flux, we next sought to identify whether ischemia-specific deficits in mitochondrial function occur in adult BALB/c mice, a parental strain with inherent susceptibility to developing myopathy after HLI relative to the adult (12–15 weeks) BL6 strain (13–17, 20–22). BALB/c mice mimic the human CLI phenotype after HLI, with reduced perfusion recovery, increased tissue loss, and exacerbated myopathy (13–17). Consistent with these previous reports, BALB/c mice displayed reduced limb perfusion recovery (Figure 4, A and B) compared with BL6 following HLI. Myopathy development was rapid in BALB/c mice, including functional deficits (isometric muscle force; Figure 4C) that were resolved quickly (HLI day 3 [d3]) in BL6 mice. Electron micrographs at HLI d7 revealed a complete disruption of sarcomere ultrastructure and mitochondrial morphology in BALB/c mice (Figure 4D). High-resolution respirometry was first performed in cage-control limb muscle mitochondria using a variety of substrate/inhibitor combinations (designed to assess multiple sites in the mitochondrial electron transport system [ETS]) to verify similar mitochondrial function at baseline (Figure 4E). Following HLI, only BALB/c limb muscle mitochondria rapidly (within 24 hours after HLI) lost respiratory function under all substrate conditions (Figure 4, F–J), suggesting complete disruption of the ETS before strain-specific limb blood flow disparities. The observed deficit, however, occurred in the absence of changes in mitochondrial content (Figure 4K). These findings establish loss of mitochondrial respiratory function as a unique strain-dependent component of ischemic BALB/c myopathy and establish the BALB/c parental strain as an ischemia inducible parental strain model for therapeutic metabolism investigation. In addition to the rapid and sustained mitochondrial impairments in ischemic BALB/c muscles, this strain, unlike BL6 mice, also suffered from a rapid loss of PFKFB3 protein abundance following HLI (Figure 4L). Moreover, primary myotubes from BALB/c mice exhibited a substantial decrease in glycolytic flux when exposed to hypoxia, whereas BL6 mice displayed a slight increase (Figure 4M). Taken together, these results suggest that ischemia-susceptible BALB/c mice experience a lack of glycolytic compensation in ischemic conditions.

### PFKFB3 gene therapy is therapeutically viable as an alternative to fragile mitochondrial function in ischemic BALB/c mice.

**Figure 5:**
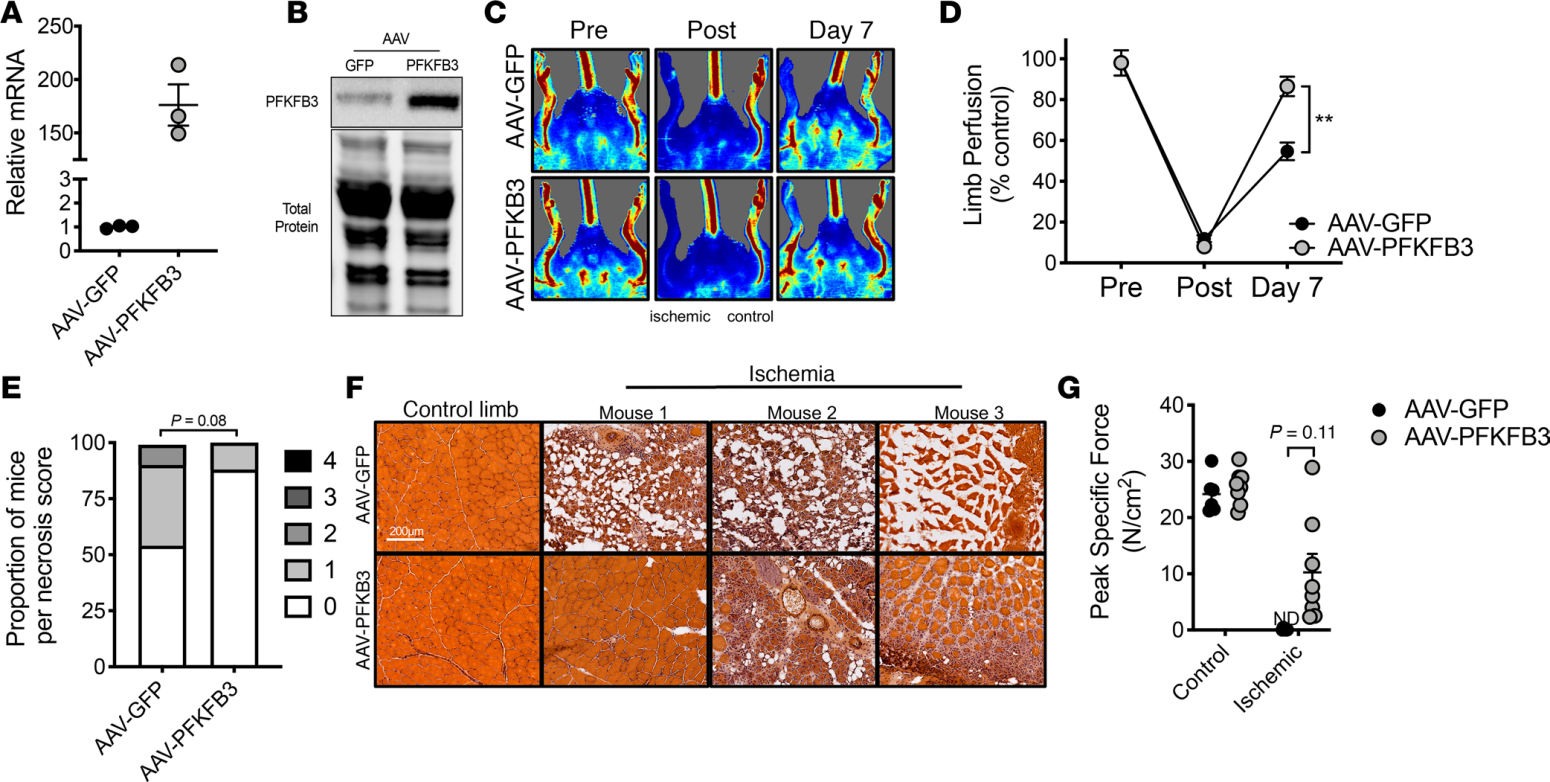
AAV-mediated expression of PFKFB3 decreases ischemic muscle necrosis and improves muscle function in BALB/c mice. BALB/c mice are known to respond poorly to hind limb ischemia, exhibiting significant muscle necrosis and — in some cases — limb loss. BALB/c mice were given intramuscular injections of AAV-GFP or AAV-PFKFB3 to the hind limb musculature at 1 × 10^11^ vg/muscle 3 weeks before HLI. (**A** and **B**) AAV expression was validated at both the mRNA (**A**; *n* = 3/group) and protein (**B**) level for PFKFB3. (**C** and **D**) AAV-PFKFB3 treatment significantly improved perfusion recovery (*n* = 10/group), measured by laser Doppler perfusion imaging. (**E** and **F**) AAV-PFKFB3–treated mice also displayed less limb necrosis (**E**, *n* = 10/group) and improved muscle histology (**F**). Scale bar: 200 µm. (**G**) Importantly, AAV-PFKFB3 also increased muscle force production, which was undetectable in all AAV-GFP mice. *n* = 10/group. **P < 0.01 versus control (AAV-GFP or DMSO) using 2-way ANOVA with Tukey’s post hoc for comparisons (**D**) and Student’s *t* test (**A** and **G**). Data in **E** were analyzed by nonparametric Mann-Whitney *U* test. Values are presented as mean ± SEM.

To explore the therapeutic efficacy of PFKFB3 in ischemic skeletal muscles, we delivered adeno-associated virus–GFP (AAV-GFP) and AAV-PFKFB3 viruses to BALB/c mice. AAV expression was validated by mRNA and protein expression of PFKFB3 (Figure 5, A and B). Mice treated with AAV-PFKFB3 demonstrated improved limb blood flow/LDPI (Figure 5, C and D) and reduced tissue loss/necrosis (Figure 5E). Histological assessment of the skeletal muscle tissue revealed a rescue of ischemic myopathic lesions with AAV-PFKFB3 (larger myofibers, more myofibers with centralized nuclei, and less nonmyofiber area) (Figure 5F) and a partial recovery of muscle force production (Figure 5G).

**Figure 6:**
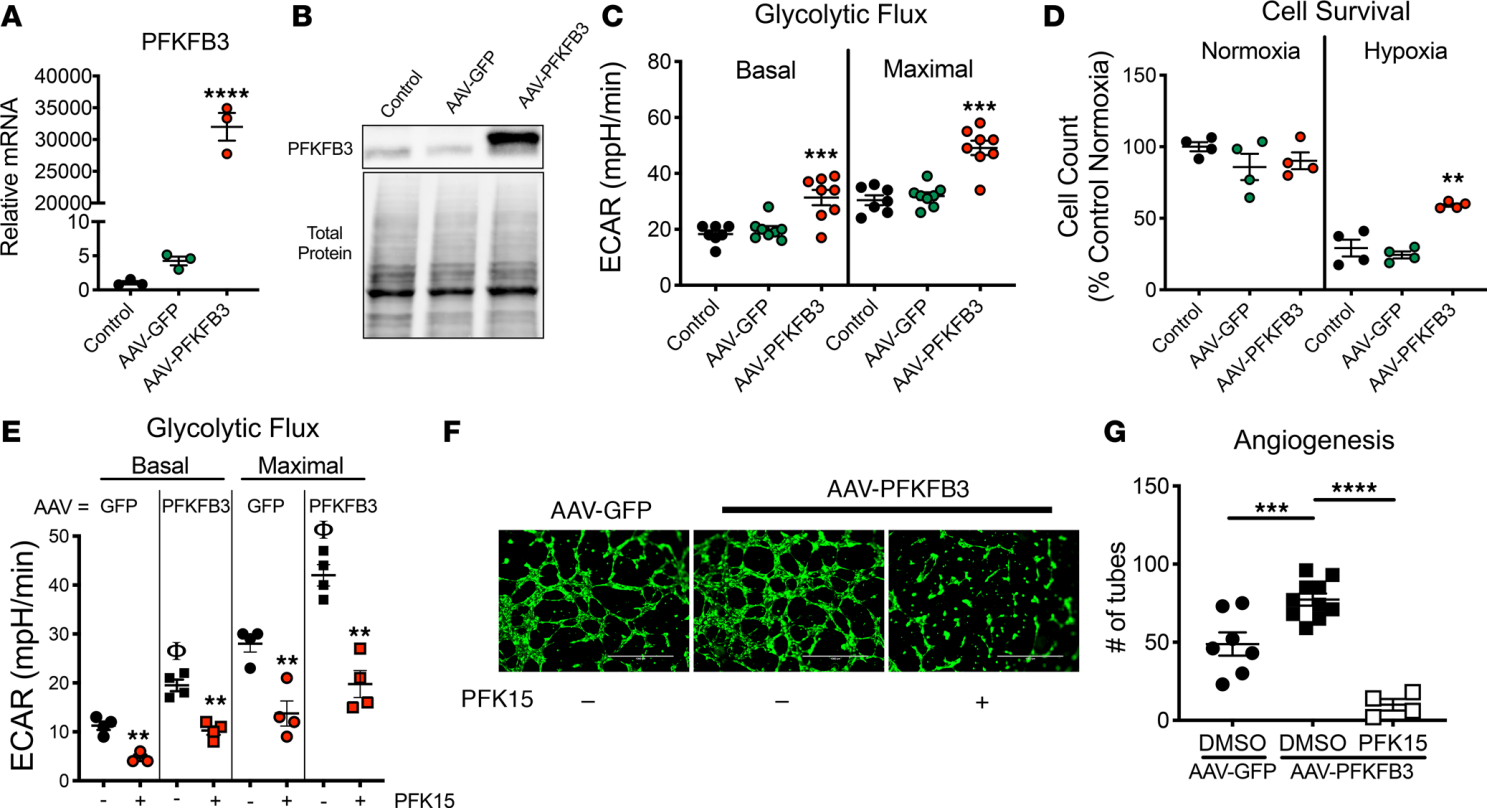
PFKFB3 expression increases glycolytic flux in skeletal muscle and endothelial cells in vitro, resulting in enhanced hypoxia tolerance and angiogenesis. Skeletal muscle cells (myotubes) and HUVECs (endothelial cells) were treated with control (AAV-GFP) and overexpression (AAV-PFKFB3) constructs. (**A** and **B**) Treatment was validated at both the mRNA (**A**, *n* = 3/group) and protein (**B**) level. (**C** and **D**) PFKFB3 overexpression increased both basal and maximal glycolytic flux in skeletal muscle cells (**C**, *n* = 7–8/group) resulting in improved cell survival/viability in hypoxia (**D**, *n* = 4/group). (**E–G**) PFKFB3 overexpression also increased both basal and maximal glycolytic flux in endothelial cells (**E** and **F**, *n* = 3–4/group), which resulted in enhanced endothelial cell tube formation (**G**, *n* = 3-4/group), an in vitro model of angiogenesis. Increased angiogenesis could be blocked by treatment with PFK15 (inhibitor of PFKFB3). **P < 0.01, ***P < 0.001, and ****P < 0.0001 versus control (AAV-GFP or DMSO) using ANOVA with Tukey’s post hoc for comparisons. ^φ^*P* < 0.05 for virus effect (**E**) using ANOVA (1-way in **A** and **G**, 2-way in **C–E**) with Tukey’s post hoc for comparisons. Values are presented as mean ± SEM.

To determine cell-specific effects of PFKFB3 overexpression, mouse muscle or human endothelial cells (HUVEC) were infected with AAV-PFKFB3 or AAV-GFP viruses (MOI of 10,000). Target expression in muscle cells was validated in both mRNA and protein analyses (Figure 6, A and B). PFKFB3 overexpression increased basal and maximal glycolytic flux rates in muscle myotubes (Figure 6C) and resulted in greater cell survival/viability in hypoxia (Figure 6D) compared with control (no virus) and AAV-GFP–treated cells. Consistent with previous findings (18), viral PFKFB3 overexpression in endothelial (HUVEC) cells increased both basal and maximal (Figure 6E) glycolytic flux, resulting in enhanced tube/mesh formation in a standard in vitro model of angiogenesis (Figure 6, F and G). Notably, these effects were abolished when cells were also treated with PFK15, a small molecule PFKFB3 inhibitor (19). Collectively, our findings highlight the therapeutic potential of PFKFB3 in both ischemic muscle and endothelial cells.

### Glycolysis in CLI patient limb muscles.

**Figure 7:**
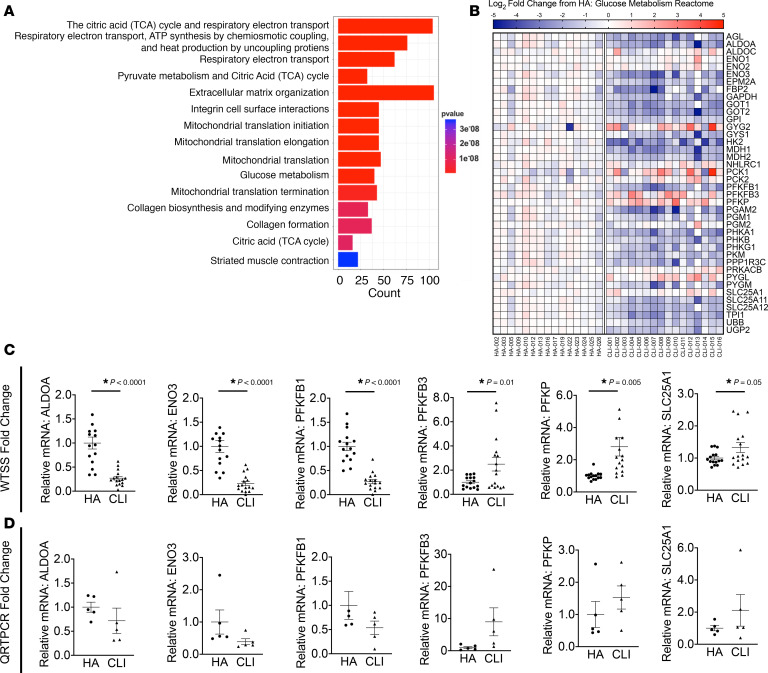
Reactome enrichment in RNA sequencing data from muscle biopsies of peripheral arterial disease patients. Gene expression profiles were determined by whole genome sequencing of RNA isolated from muscle biopsy samples of the gastrocnemius. (**A**) Reactome enrichment analysis indicated that the most significant gene expression changes were related to bioenergetics (including mitochondria and glucose metabolism). (**B**) A heatmap of gene expression differences (log_2_ [fold change from healthy adult (HA)]) for the Reactome terms and genes associated with glucose metabolism. (**C**) mRNA fold-changes from HA in WTSS data set used for Reactome enrichment. (**D**) mRNA directional changes were validated by qPCR of selected genes. Statistical testing performed in **C** using 2-tailed Student’s *t* test. Values are presented as mean ± SEM.

**Figure 8:**
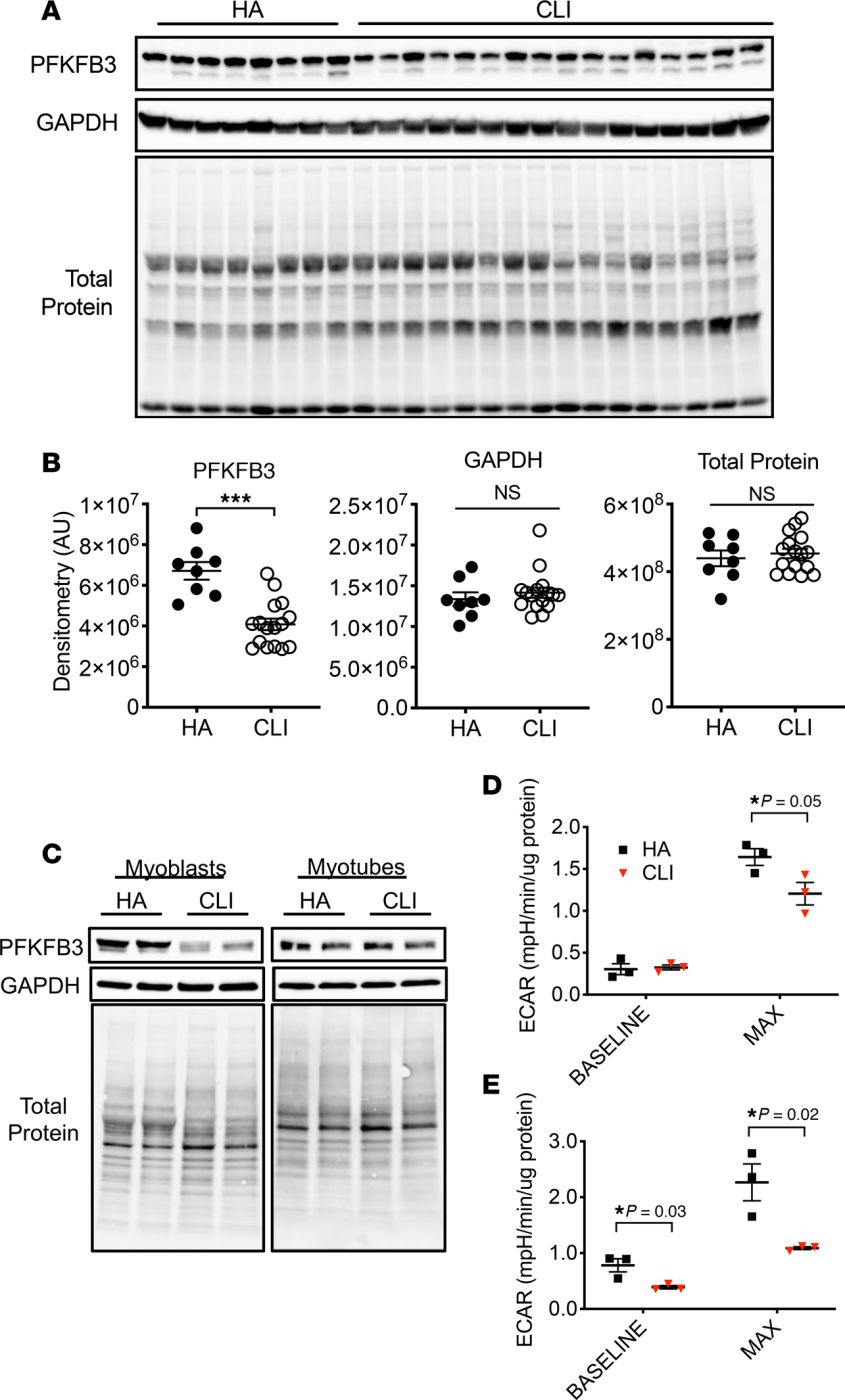
PFKFB3 protein and muscle progenitor cell glycolytic flux are reduced in CLI patients. Western blotting was performed on gastrocnemius muscle lysates from age-matched healthy adults without PAD (*n* = 8) and severe PAD patients with critical limb ischemia (CLI, *n* = 16). (**A**) Western blot images for PFKFB3, GAPDH, and total protein on PVDF membrane. (**B**) Quantified densitometry of images in **A**. Western blotting was performed on primary muscle cell myoblast and differentiated myotube lysates from age-matched healthy adults without PAD (HA; *n* = 3) and severe PAD patients with critical limb ischemia (CLI, *n* = 3). (**C**) Western blot images for PFKFB3, GAPDH, and total protein on PVDF membrane. (**D** and **E**) Quantified extracellular acidification rate (Seahorse) assay under baseline and maximal stimulation conditions in myoblasts (**D**) and differentiated myotubes (**E**). ***P < 0.001 using 2-tailed Student’s *t* test. Values are presented as mean ± SEM.

We recently identified a unique and severe mitochondriopathy in human CLI patient limb muscle tissues defined by reduced mitochondrial oxidative capacity, reduced ETS enzyme function, decreased abundance of mitochondrial-associated mRNAs and proteins, and a corresponding retention of the altered transcriptional program and mitochondrial function in isolated primary muscle cells from these patients (5). With this in mind, and using the same set of patients, we next determined (a) whether CLI patient tissues fail to upregulate glycolysis and PFKFB3 as an adaptation to reduced oxidative phosphorylation and (b) whether glycolysis is upregulated in the muscle cells of CLI patients. Reactome enrichment of our previously published RNA-seq data indicated that the most significant gene expression changes were related to bioenergetics (including mitochondria and glucose metabolism; Figure 7A). A heatmap for the glucose metabolism Reactome revealed unique expression patterns for this category in CLI patients, largely defined by reductions in mRNA (Figure 7B), which we then validated for a series of select targets using fold-change values from the whole transcriptome shotgun sequencing (WTSS) data set (Figure 7C) already published (5). Then, using a few available samples from the same patients, we validated directionality using quantitative PCR (qPCR) for the same targets (Figure 7D). Lysates generated from the skeletal muscles of CLI patients displayed decreased PFKFB3 expression in limb muscle biopsy samples compared with non-PAD control subjects (Figure 8, A and B). Isolated primary CLI myoblasts mirrored this deficit in vitro, although differentiation into myotubes resulted in a similar abundance of PFKFB3 protein to healthy control primary myotubes (Figure 8C). Regardless of the abundance of PFKFB3 protein, primary myoblasts and myotubes generated from CLI patients demonstrated reductions in glycolytic flux (ECAR) compared with healthy adult cells (Figure 8, D and E). These findings demonstrate a lack of glycolytic compensation for deficient mitochondrial function in CLI patient primary muscle cells.

## Discussion

While exploring the role of mitochondrial sufficiency in ischemic limb myopathy, this study revealed that enhanced glycolytic metabolism in homozygous aged mtDNA mutator mice provided remarkable protection from ischemic muscle injury in a murine model of PAD. This metabolic switch was at least in part driven by expression of PFKFB3, a recognized mediator of heightened glucose fermentation in cancer cells (23). We also revealed that adult BALB/c mice, which are known to respond poorly to limb ischemia (7, 13–15, 17), exhibited a rapid and severe loss of muscle mitochondrial respiratory function after HLI onset. Unlike mtDNA mutator mice, however, BALB/c mice did not respond by increasing PFKFB3. Therapeutically, AAV-mediated delivery of PFKFB3 significantly improved limb perfusion recovery and partially rescued muscle contractile function after HLI onset in these mice. Overall, our preclinical findings reveal that enhancement of glycolytic flux, during periods of reduced blood flow and/or under conditions of mitochondrial dysfunction, is capable of satiating the bioenergetic demands of ischemic limb muscle and improving myopathy. Clinically, Reactome and lysate analysis in muscles from CLI patients verified a reduction in targets related to glucose metabolism. Primary muscle cells from these patients possessed attenuations in glycolytic flux as both myoblasts and differentiated myotubes. These findings are striking, given that these patients (and cells) also demonstrate significant reductions in mitochondrial function (5). Collectively, this suggests a potentially devastating inadequacy in muscle metabolic flexibility in patients with the most severe manifestation of PAD.

The PFKFB family of enzymes function to phosphorylate fructose-6-phosphate to fructose-2,6-bisphosphate. All PFKFB proteins exhibit bifunctional activity capable of both kinase and phosphatase reactions. PFKFB3 is unique because it maintains a much higher preference for kinase activity, resulting in nearly unidirectional production of fructose-2,6-bisphosphate and is highly expressed in skeletal muscle (24). Fructose-2,6-bisphosphate, in-turn, activates 6-phosphofructo-1-kinase (a rate-limiting enzyme in glycolysis). For these reasons, PFKFB3 has been recognized as an important regulator of glycolytic metabolism (18, 25). Many cancerous cells and tumors exhibit increased expression of PFKFB3 (23), leading to the development of chemical inhibitors aimed to treat cancer (19). The ability of cells to rapidly switch metabolic pathways (aerobic-to-glycolytic) has been a defining characteristic of cancerous cells that enables survival within a hypoxic tumor environment (26, 27). This plasticity represents an evolutionary adaptation, which, while not necessarily desirable in the context of tumor development, provides an avenue for therapeutic leverage in instances of pathologic ischemic necrosis.

Ischemia imposes a major energetic challenge on cells due to impaired oxidative phosphorylation, which can lead to a decrease in cellular energy charge, disruption of ion homeostasis, and necrotic cell death. Organisms/tissues that are capable of the necessary plasticity to use both oxidative and glycolytic ATP production efficiently, based on substrate and oxygen availability in the local environment, are at a distinct advantage in ischemic pathology manifestation. This is particularly evident in the overall context of the metabolic capacities of the mice in this study. The muscle mitochondria of adult BL6 and BALB/c mice are similar under baseline conditions. While suffering equally severe reductions in limb blood flow with HLI, they demonstrate individualized mitochondrial responses in the postsurgery recovery week. For BL6 mice, the ability of their mitochondria to withstand the local environment during the initial dip in limb blood flow undoubtedly provides a distinct advantage during recovery. mtDNA mutator mice, which are bred in the BL6 background, are not afforded the same ischemic mitochondrial superiority as the BL6 parental strain. These homozygous mice also suffer from progeria. In our studies, aging both the WT and heterozygous littermates resulted in more severe ischemic phenotypes from commonly used adult (12- to 15-week) BL6 mice. Aged D257A^+/+^ mice, however, are unique in their ability to efficiently compensate via priming of their glycolytic capacity in response to an accelerated lifetime of mitochondrial fragility. This finding is supported by a recently published proteomic screening in slightly younger mtDNA mutator male mice (28). In the case of BALB/c mice, muscle mitochondria wilt during the same acute ischemia phase of HLI, and they are incapable of shifting efficiently to glycolytic flux, resulting in catastrophic effects on the limb. This is particularly interesting considering that these mice are generally used during adulthood (approximately 12–15 weeks old), before any influence of senescence. The beneficial effects of a gene therapy approach (AAV-PFKFB3) to improve limb perfusion and muscle function in BALB/c mice supports the viability of driving glycolytic flexibility as a therapeutic option. This is particularly prescient in the context of CLI patients, whose muscle cells harbor a unique mitochondrial fragility and parallel decreases in glycolytic flux in vitro. Enhanced glycolysis protects the myocardium during ischemia (29–31) and our findings collectively support the efficacy of this therapeutic angle for diseases involving ischemic skeletal muscles.

Together, our data raise an intriguing question: Is the protective effect of PFKFB3 mediated primarily through its vascular/angiogenic effects or through its impact on skeletal muscle cell metabolism? Recent work revealed a pivotal role of PFKFB3 expression in endothelial cell sprouting and angiogenesis (18, 32). Specifically, genetic knockdown or chemical inhibition of PFKFB3 reduced angiogenesis both in vitro and in vivo, whereas overexpression was shown to increase vessel sprouting (18, 33, 34). Herein, we confirmed these proglycolytic and proangiogenic effects of viral overexpression of PFKFB3 in endothelial cells, but we also observed enhanced muscle cell survival in hypoxia. Furthermore, PFKFB3 gene therapy in mice was driven by the ubiquitous CMV gene promoter, which may have imparted a benefit in both muscle and endothelial cells within the ischemic limb. While future studies are needed to further dissect the cell-specific effects of PFKFB3 and each cell’s respective impact on ischemic pathology, there is clear preclinical evidence to support both therapeutic approaches. Many cell- and gene-based angiogenic therapies exhibit positive effects on limb pathology in preclinical animal studies; however, many of these therapies lack strong clinical/translational efficacy to date (reviewed in refs. 35, 36). Recent evidence from our group and others lends support to the hypothesis that the pathobiology of limb ischemia is mediated not solely by tissue perfusion, but by other tissues such as skeletal muscle, which play a critical role in both preclinical and clinical phenotypes (6, 12, 14–17, 22, 37–41). In our study, several components point to the importance of the skeletal muscle cells in this context, including: (a) that there is no expansion of limb blood flow (LDPI) or capillary perfusion (Lectin^+^ vessel area) in D257A^+/+^ limb skeletal muscles at baseline (as compared with D257A^+/–^ or WT^–/–^ mice); (b) the ischemia-related (HLI) alterations in BALB/c mitochondrial function occur prior (d1) to realized deficits in limb blood flow (LDPI) versus BL6; (c) the AAV9 serotype used in vivo in our studies is recognized for its efficiency in targeting skeletal muscle (42); and (d) overexpression of PFKFB3 in primary muscle cells in vitro mimicked the observed beneficial response of intervention on myopathy in vivo.

In the local limb environment, an intriguing scenario is that PFKFB3 is having beneficial effects on both the muscle and vascular cells, where the combined effects have a greater physiological impact within the ischemic limb. The metabolic demand of skeletal muscle myofibers is a key driver of alterations in limb muscle perfusion (43). A metabolically fragile and myopathic limb microenvironment is not likely to immediately provide sufficient bioenergetic support to halt an ischemic pathologic slide, even with an acute restoration of blood flow. Additionally, this microenvironment is not tailored to support the stabilization of nascent collateral vessels or vascular grafts, a concept supported by the fact that surgical intervention in CLI patients often results in limb amputation or patient death, despite graft patency and/or improvements in arterial flow by angiography (44–47). The temporal survival, initiation of regeneration, and release of muscle-derived vascular growth factors may be critical to proper support of vascular networks in the PAD (48). Thus, a critical step to identifying effective therapies is to gain a thorough understanding of the metabolic capacity and biology of multiple cell types in the critically chronic ischemic limb of patients.

This work suggests an interesting paradigm: enhanced ischemic limb muscle glycolysis is sufficient to support survival during the transient early window of insult. Work by Aragonés et al. (49) provides direct evidence of the efficacy of metabolic reprogramming toward anaerobic glycolysis as a therapeutic avenue to prevent myopathic outcomes after HLI. Specifically, loss of the HIF prolyl hydroxylase Phd1 results in an adaptive stimulation of muscle glycolysis at baseline, which in turn protects the skeletal muscle from ischemic insult during the early phase of HLI. This adaptation mirrors the glycolytic shift and myopathic resistance seen in our D257A^+/+^ mouse studies and provides strong complementary evidence for therapeutic development in this space, which took the form of PFKFB3 gene therapy in our studies. The period of ischemic injury that occurs before restoration of limb flow and capillary perfusion is often overlooked but is largely represented by the time preceding HLI d3 preclinically (3, 4, 6, 50). During this temporal window, locally severely hypoxic or anoxic conditions logically limit the oxidation of fatty acids or carbohydrates and likely render the skeletal muscle mitochondria stagnant or dysfunctional. If overall ATP production fails to meet basic cellular needs, it is likely that irreversible alterations in cellular osmolar load, ionic imbalance, and membrane disruption will occur. In the case of PAD, our collective work suggests that this is a probable determinant of ultimate clinical presentation (claudicating versus chronic limb threatening ischemia). Tightly regulated supplementary glycolysis as a therapeutic avenue has gained traction in other ischemic disease model systems, including liver (51) and cardiac muscle (52). A similar approach aimed at developing glycolytic therapies for PAD patients to prevent an inevitable march to the myopathic “tipping point” warrants additional investigation. A logical merger for effective therapeutic implementation would likely involve both glycolytic stimulation and long-term support for the mitochondrial network.

### Conclusion.

In summary, the current study has provided evidence demonstrating that enhanced glycolytic metabolism, mediated at least in part by expression of PFKFB3, confers protection from ischemic muscle injury in mice following HLI. The enhanced glycolytic metabolism has numerous favorable effects: increased limb perfusion, sustained capillary density, decreased muscle necrosis, and improved muscle contractile function. These findings are of great clinical relevance, as limb muscle tissues from CLI patients display alterations in the glucose Reactome and reductions in PFKFB3 protein. Furthermore, primary muscle cells from CLI patients possess an inherent deficit in glycolytic capacity. These results support the premise that metabolic health is crucial in disease manifestation and suggest that glycolytic flexibility may serve as a novel therapeutic target for PAD — and, specifically, CLI.

## Methods

Supplemental Methods are available online with this article (https://doi.org/10.1172/jci.insight.139628DS1).

### Study participants.

Twenty-six healthy adults without PAD (HA) and 19 patients with CLI were recruited through print advertising or identified by vascular surgeons at East Carolina University Brody Medical Center. Inclusion criteria consisted of patients with CLI undergoing amputation. Exclusion criteria consisted only of CLI amputation patients who previously provided biological specimens from the contralateral limb. All data collection was carried out by blinded investigators at East Carolina University. Detailed patient information for this cohort has previously been published (5).

### Animals.

Heterozygous Polg mtDNA mutator (D257A^+/–^) breeders (stock no. 017341), BL6 (stock no. 000664), and BALB/c mice (stock no. 000651) were obtained from The Jackson Laboratory. D257A**^+/–^** mice (*n* = 90) were bred to generate D257A^–/–^ (WT), D257A^+/–^, and D257A^+/+^ littermates and genotyped according to instructions from the Jackson Laboratory. Experimental D257A mice were used at 12 months of age. For strain HLI studies, male BL6 (*n* = 40) and BALB/c (*n* = 40) mice were used at 12 weeks of age. For AAV studies, male BALB/c mice (*n* = 20) were used at 12 weeks of age. AAVs were locally delivered via intramuscular injections of the hind limb musculature (plantarflexors and dorsiflexors) at 5 × 10^10^ vg/injection site 2 weeks before HLI. All rodents were housed in a temperature (22°C) and light-controlled (12-hour light/12-hour dark) room and maintained on standard chow with free access to food and water. HLI, necrosis scoring, LDPI were performed as previously described (3, 6, 12, 17).

### Mitochondrial isolation and functional assays.

Skeletal muscle mitochondria were isolated from the plantar flexor (i.e., gastrocnemius, soleus, and plantaris) muscles of both control and ischemic limbs, as previously described (12). High-resolution O_2_ consumption measurements were performed to assess respiratory function, as previously described (3, 12). Citrate synthase activity was measured spectrophotometrically, as previously described (53).

### Murine tissue RNA isolation and transcriptome sequencing.

RNA-seq was performed by Quick Biology Inc. RNA integrity was checked by Agilent Bioanalyzer 2100; only samples with clean rRNA peaks were used. The library for RNA-seq data was prepared according to KAPA Stranded mRNA-seq poly(A) selected kit with 201–300 bp insert size (KAPA Biosystems) using 250 ng total RNA as input. Final library quality and quantity was analyzed by Agilent Bioanalyzer 2100 and Life Technologies Qubit3.0 Fluorometer. Paired end reads (150 bp) were sequenced on Illumina HighSeq 4000 (Illumina Inc.). The reads were first mapped to the latest UCSC transcript set using STAR version 2.4.1d, and the gene expression level was quantified to annotation model (Partek E/M). Gene expression levels were normalized using trimmed mean of M-values (TMM). Differentially expressed genes were identified using ANOVA in Partek. Genes showing altered expression with FDR < 0.05 and more than 2-fold changes were considered differentially expressed. GOseq was used to perform the GO enrichment analysis, and KOBAS was used to perform the pathway analysis. Heatmaps were generated with Pheatmap program using a log_2_ (fold change from WT control).

### Skeletal muscle morphology and function.

Skeletal muscle morphology and vessel density were assessed by standard light microscopy and IF microscopy. Transverse sections (10 μm thick) from TA were cut using a cryotome and collected on charged slides for staining. For morphological analyses, standard methods for H&E histological staining were performed, and images were obtained using an Evos FL Auto microscope (Thermo Fisher Scientific). Perfused capillaries were measured in vivo by labeling endothelial cells with Dylight594 conjugated Griffonia simplicifolia I isolectin B4 (Vector Labs) injected retro-orbitally in anesthetized mice. All image analysis was conducted by a blinded investigator. Skeletal muscle contractile function was assessed in the EDL muscle, as previously described (12, 17).

### AAV generation.

PFKFB3 was PCR amplified from BALB/c genomic DNA and inserted into an AAV-CMV cloning vector (generated in house) using In-Fusion cloning reagents (Takara). AAVs for GFP and PFKFB3 were generated by triple transfection of Hek293T cells (with AAV9 and pHelper plasmids from Cell Biolabs) and purified using purification kits from Takara (catalog 6666). AAVs were locally delivered via intramuscular injections of the hind limb musculature (plantarflexors and dorsiflexors) at 5 × 10^10^ vg/injection site 2 weeks before HLI.

### Human tissue RNA isolation and transcriptome sequencing.

Details and full results of the RNA-seq analysis are previously published and available online (5). The data were also previously deposited in NCBI’s Gene Expression Omnibus (GEO) and can be accessed using GEO Series accession number GSE114070. Total RNA was extracted using QIAGEN RNeasy Midi kits per manufacturer instructions. RNA-seq was performed by Quick Biology Inc. RNA integrity was checked by Agilent Bioanalyzer 2100; only samples with clean rRNA peaks were used. The library for RNA-seq data was prepared according to KAPA Stranded mRNA-seq poly(A) selected kit with 201–300 bp insert size (KAPA Biosystems) using 250 ng total RNA as input. Final library quality and quantity was analyzed by Agilent Bioanalyzer 2100 and Life Technologies Qubit3.0 Fluorometer. Paired end reads (150 bp) were sequenced on Illumina HighSeq 4000 (Illumina Inc.). The reads were first mapped to the latest UCSC transcript set using Bowtie2 version 2.1.0 (54), and the gene expression level was estimated using RSEM v1.2.15 (55). TMM was used to normalize the gene expression. Differentially expressed genes were identified using the edgeR program (56). Genes showing altered expression with *P* < 0.05 and more than 1.5-fold changes were considered differentially expressed. GOseq was used to perform the GO enrichment analysis, and Reactome was used to perform the Reactome analysis specific for this work. Heatmaps were generated with Prism using a log_2_ (fold change from non-PAD) of the glucose metabolism Reactome. To further validate RNA-seq findings, RNA was reverse transcribed using Superscript IV Reverse Transcriptase according to manufacturer instructions (Invitrogen). Real-time PCR on selected gene targets was performed using a Quantstudio 3 Real-time PCR system (Applied Biosystems). Relative quantification of mRNA levels was determined using the comparative threshold cycle (ΔΔCt) method using FAM-labeled TaqMan Gene expression assays (Applied Biosystems) specific to the given gene run in multiplex with a VIC-labeled 18s control primer.

### Primary muscle progenitor cell isolation, cell lines, and culture.

Primary human muscle precursor cells (human myoblasts) were derived from fresh muscle biopsy samples, as previously described (5). Primary muscle progenitor cells (MPC) were also isolated and cultured from BALB/c mice, as previously described (3, 14). Primary MPCs (human or mouse) were plated at 150,000 cells/well into Seahorse XF24 assay plates coated with entactin/collagen/laminin (EMD Millipore) and were allowed to adhere overnight, differentiated by serum withdrawal for 5 days (DMEM + 2% horse serum). HUVECs were seeded at 100,000 cells/well on 0.1% gelatin coated dishes. For AAV experiments, BALB/c primary muscle cells were seeded into XF24 assay plates at confluence; infected with AAV at MOI of 10,000; and differentiated via serum withdrawal (DMEM + 2% horse serum) for 5 days. Extracellular acidification rate, a measure of glycolytic flux, was assessed using a Seahorse XF24 machine.

### Statistics.

Data are presented as mean ± SEM. Comparisons between 2 groups were performed by Student’s 2-tailed *t* test. Comparisons of data with more than 2 groups were performed using 2-way ANOVA with Tukey’s post hoc multiple comparisons. Repeated-measures ANOVA was performed when appropriate. Nonparametric Mann-Whitney *U* testing was used to determine differences between the distributions of necrosis scores between groups. All statistical analyses were performed in GraphPad Prism (Version 6.0) or Vassarstats (www.vasserstats.net) unless otherwise specified. In all cases, *P* < 0.05 was considered statistically significant.

### Study approval.

This study was approved by the IRB at East Carolina University and carried out in accordance with the Declaration of Helsinki. All participants gave written informed consent. All animal experiments adhered to the *Guide for the Care and Use of Laboratory Animals* (National Academies Press, 2011). All procedures were approved by the IACUC of East Carolina University.

## Author contributions

JMM had full access to the data presented and takes responsibility for the integrity and accuracy of data. JMM, TER, and DJY were responsible for conception and design of the study. JMM, TER, DJY, CAS, AO, JME, TDG, MDT, EJG, MMRI, RK, KFW, PDN, AJA, and EES were responsible for data acquisition, analysis, and/or interpretation. TER, DJY, and JMM drafted the manuscript. TER, DJY, AO, JME, PDN, EES, KFW, and JMM edited and revised the manuscript. JMM obtained funding. TER, DJY, CAS, PB, TDG, MDT, RK, AO, JME, PDN, KFW, EES, and JMM provided administrative, technical, or material support.

## Supplementary Material

Supplemental data

## Version 1 08/25/2020

In-Press Preview

## Version 2 09/17/2020

Electronic publication
