# Supplementary material for: PFKFB3-mediated glycolysis rescues myopathic outcomes in the ischemic limb
Source: JCI Insight. 2020 Sep 17;5(18):e139628. doi: 10.1172/jci.insight.139628 (PMC7526546; doi:10.1172/jci.insight.139628)
Supplement: Supplemental data [file jciinsight-5-139628-s159.pdf]

PFKFB3 mediated glycolysis rescues myopathic outcomes in the ischemic limb.

Terence E. Ryan[1,2], Cameron A. Schmidt[1,2], Michael D. Tarpey[1,2], Adam J. Amorese[1,2], Dean J. Yamaguchi[3,4], Emma J. Goldberg[1,2], Melissa M.R. Iñigo[1,2], Reema Karnekar[1,2], Allison O'Rourke[5], James M. Ervasti[5], Patricia Brophy[1], Thomas D. Green[1,2], P.D. Neufer[1,2], Kelsey Fisher-Wellman[1,2], Espen E. Spangenburg[1,2], Joseph M. McClung[1,2,3,#].

[1]East Carolina Diabetes and Obesity Institute, [2]Department of Physiology, [3]Department of Cardiovascular Science, [4]Division of Surgery, East Carolina University, Brody School of Medicine, Greenville, NC.
[5]Department of Biochemistry, Molecular Biology and Biophysics, College of Biological Sciences, University of Minnesota, Saint Paul, MN.

Supplementary Materials

Running Title: *Ryan et al.*; PFKFB3 rescues ischemic myopathy

#Correspondence should be addressed to J.M.M.: Diabetes and Obesity Institute, Office #4109, Mail Stop 743, East Carolina Heart Institute, Brody School of Medicine at East Carolina University, 115 Heart Drive, Greenville, NC 27834-4354. Tel: 252-737-5034 (office); Fax: 252-744-3460; email: mcclungj@ecu.edu

Keywords: ischemia, peripheral artery disease, mouse genetics, mitochondria, skeletal muscle regeneration

Supplementary Methodology

Animals. Heterozygous Polg mtDNA mutator (D257A$^{+/-}$) breeders (Stock # 017341), C57BL6J (Stock #000664), and BALB/cJ mice (Stock # 000651) were obtained from The Jackson Laboratory. D257A$^{+/-}$ mice (*N*=90) were bred to generate D257$^{-/-}$ (WT), D257$^{+/-}$, and D257$^{+/+}$ littermates and genotyped according to instructions from Jackson Labs. Experimental D257 mice were used at 12-months of age. For strain HLI studies, male C57BL6J (n=40) and BALB/cJ (n=40) mice were used at 12-weeks of age. For AAV studies, male BALB/cJ mice (n = 20) were used at 12-weeks of age. Adeno-associated viruses (AAVs) were locally delivered via intramuscular injections of the hindlimb musculature (plantarflexors and dorsiflexors) at 5 x 10^{10} vg/injection site two weeks prior to HLI. All rodents were housed in a temperature (22˚C) and light controlled (12-hour light/12-hour dark) room and maintained on standard chow with free access to food and water. Hindlimb ischemia, necrosis scoring, laser Doppler perfusion imaging (LDPI) were performed as previously described(4,6,10,11). All animal experiments adhered to the Guide for the Care and Use of Laboratory Animals from the Institute for Laboratory Animal Research, National Research Council, Washington, D.C., National Academy Press, 1996, and any updates. All procedures were approved by the Institutional Animal Care and Use Committee of East Carolina University.

Human Study Oversight. This study was approved by the institutional review boards at East Carolina University (ECU), the University of Nebraska Medical Center, and Veterans Affairs Nebraska-Western Iowa and carried out in accordance with the Declaration of Helsinki. All participants gave written informed consent.

Human Study Participants. Detailed patient information for this cohort has previously been published(1). Twenty-six healthy adults without PAD (HA) and nineteen patients with critical limb ischemia (CLI) were recruited through print advertising or identified by vascular surgeons at East Carolina University Brody Medical Center. Exclusion criteria consisted only of CLI amputation patients who previously provided biological specimens from the contralateral limb. All data collection was carried out by blinded investigators at ECU.

Tissue Acquisition. Percutaneous muscle biopsy samples were taken from the lateral gastrocnemius muscle of age-matched volunteer HA and identified PAD patients. The skin of the muscle biopsy sight was first cleansed with a povidone-iodine swab and then anesthetized with 5 cc of lidocaine. A small incision was made and the muscle biopsy sample was aspirated through a 5 mm Bergstrom needle. Tissue obtained from CLI patients occurred inside the operating room immediately following limb amputation performed without the use of a tourniquet. Muscle specimens from CLI patients were collected form the same anatomical location of the gastrocnemius muscle as the percutaneous muscle biopsies performed in HA and IC patients. Part of the muscle biopsy sample was immediately flash frozen in liquid nitrogen and stored at -80°C until subsequent analysis. Other portions of the muscle biopsy (~80-150 mg) were used for human primary muscle cell isolation and permeabilized myofiber mitochondrial experiments.

Human tissue RNA isolation and transcriptome sequencing. Details and full results of the RNA sequencing analysis are previously published and available online(1). The data were also previously deposited in NCBI's Gene Expression Omnibus (GEO) and

can be accessed using GEO Series accession number GSE114070. Total RNA was extracted using Qiagen RNeasy Midi kits per manufacturer instructions. RNA sequencing was performed by Quick Biology Inc. (Pasadena, CA, USA). RNA integrity was checked by Agilent Bioanalyzer 2100; only samples with clean rRNA peaks were used. Library for RNA-Seq was prepared according to KAPA Stranded mRNA-Seq poly(A) selected kit with 201-300bp insert size (KAPA Biosystems, Wilmington, MA) using 250 ng total RNA as input. Final library quality and quantity was analyzed by Agilent Bioanalyzer 2100 and Life Technologies Qubit3.0 Fluorometer. 150 bp paired end reads were sequenced on Illumina HighSeq 4000 (Illumnia Inc., San Diego, CA). The reads were first mapped to the latest UCSC transcript set using Bowtie2 version 2.1.0(13) and the gene expression level was estimated using RSEM v1.2.15.(14) TMM (trimmed mean of M-values) was used to normalize the gene expression. Differentially expressed genes were identified using the edgeR program. Genes showing altered expression with $p < 0.05$ and more than 1.5-fold changes were considered differentially expressed. Goseq was used to perform the GO enrichment analysis and Reactome was used to perform the reactome analysis specific for this work. Heatmaps were generated with Prism using a $\log_2$ [Fold Change from Non-PAD] of the Glucose Metabolism Reactome. To further validate RNA-seq findings, RNA was reverse transcribed using Superscript IV Reverse Transcriptase according to manufacturer instructions (Invitrogen). Real-time PCR on selected gene targets was performed using a Quantstudio 3 Real-time PCR system (Applied Biosystems, Foster City, CA). Relative quantification of mRNA levels was determined using the comparative threshold cycle ($\Delta\Delta$CT) method using FAM-labeled Taqman® Gene

expression assays (Applied Biosystems) specific to the given gene run in multiplex with a VIC-labeled 18s control primer.

Mouse RNA isolation and transcriptome sequencing. Total RNA was extracted using Qiagen RNeasy Midi kits per manufacturer instructions. RNA sequencing was performed by Quick Biology Inc. (Pasadena, CA, USA). RNA integrity was checked by Agilent Bioanalyzer 2100; only samples with clean rRNA peaks were used. Library for RNA-Seq was prepared according to KAPA Stranded mRNA-Seq poly(A) selected kit with 201-300bp insert size (KAPA Biosystems, Wilmington, MA) using 250 ng total RNA as input. Final library quality and quantity was analyzed by Agilent Bioanalyzer 2100 and Life Technologies Qubit3.0 Fluorometer. 150 bp paired end reads were sequenced on Illumina HighSeq 4000 (Illumnia Inc., San Diego, CA). The reads were first mapped to the latest UCSC transcript set using Bowtie2 version 2.1.0 and the gene expression level was estimated using RSEM v1.2.15. TMM (trimmed mean of M-values) was used to normalize the gene expression. Differentially expressed genes were identified using the edgeR program. Genes showing altered expression with $p < 0.05$ and more than 1.5-fold changes were considered differentially expressed. Goseq was used to perform the GO enrichment analysis and Kobas was used to performed the pathway analysis. Heatmaps were generated with Pheatmap program using a $\log_2$ [Fold Change from Non-PAD]. To further validate RNA-seq findings, RNA was reverse transcribed using Superscript IV Reverse Transcriptase according to manufacturer instructions (Invitrogen). Real-time PCR on selected gene targets was performed using a Quantstudio 3 Real-time PCR system (Applied Biosystems, Foster City, CA). Relative quantification of mRNA levels was determined using the comparative threshold cycle

(ΔΔCT) method using FAM-labeled Taqman® Gene expression assays (Applied Biosystems) specific to the given gene run in multiplex with a VIC-labeled 18s control primer.

Reagents. The following commercial antibodies were used: PFKFB3 (ProteinTech #13763), dystrophin (Thermo Scientific RB-9024), and GAPDH (Thermo Scientific MA5-15738). DAPI mounting medium (VECTOR Laboratories, H-1200) was used to coverslip slides. Secondary anti-bodies for immunofluorescence imaging were Alexa Fluor-568 (Invitrogen A21134). Histological stains were obtained from Sigma-Aldrich: Mayer's hematoxylin (Sigma MHS1) and eosin (Sigma HT110132-1L). PFK15 was obtained from EMDmillipore (#531005). All other chemicals were obtained from Sigma-Aldrich.

Adeno-associated virus generation. PFKFB3 was PCR amplified from BALB/c genomic DNA and inserted into an AAV-CMV cloning vector (generated in house) using In-Fusion cloning reagents (Takara). Adeno-associated viruses for GFP and PFKFB3 were generated by triple transfection of Hek293T cells (with AAV-DJ and pHelper plasmids from Cellbiolabs) and purified using purification kits from Takara (Cat # 6666). AAVs were locally delivered via intramuscular injections of the hindlimb musculature (plantarflexors and dorsiflexors) at 5 x 10^{10} vg/injection site two weeks prior to HLI.

Animal models of ischemic peripheral artery disease. Acute hindlimb ischemia (HLI)(2) was performed by anesthetizing mice with intraperitoneal injection of ketamine (90 mg/kg) and xylazine (10 mg/kg) and surgically inducing unilateral hindlimb ischemia by ligation and excision of the femoral artery from its origin just below the inguinal ligament. The inferior epigastric, lateral circumflex, and superficial epigastric artery

branches of the femoral artery were left intact, thereby preserving collateral perfusion to the limb. The extent of necrosis, if any, was recorded using the previously described semi-quantitative scale(2): grade 0, no necrosis in ischemic limb; grade I, necrosis limited to the toes; grade II, necrosis extending to the dorsum pedis; grade III, necrosis extending to crus; grade IV, necrosis extending to mid-tibia or complete limb necrosis.

Assessment of limb perfusion. Laser Doppler perfusion image (LDPI) scanning was performed using a Moor Instruments LDI2-High Resolution (830nM) System (Moor, Axminster, UK). Limb hair was removed bilaterally prior to scanning. Imaging was performed at 4 ms/pixel scan rate with animals placed on a 37°C warming pad under ketamine/xylazine anesthesia. Image quantification was performed by manual selection of polygonal regions of interest selected by anatomical position followed by quality assessment and export of mean flux values for statistical analysis.

Assessment of perfused capillary density. Perfused capillaries were measured by *in vivo* labeling with Dylight594 conjugated Griffonia simplicifolia I isolectin B4 (VectorLabs). Mice were anethesized with isoflurane and received a 50ul retro-orbital injection of the fluorescent lectin. Mice were returned to their cages to resume normal activity for two hours to allow systemic delivery of lectin prior to sacrifice. The TA muscle was frozen in liquid nitrogen cooled isopentane, cryosectioned at 10μm, and counterstained with dystrophin and DAPI. Perfused capillary density was assessed as an area of lectin positive capillaries per field of view. Images were manually thresholded and analyzed using Image J by a blinded investigator.

Skeletal muscle isometric force production. Contractile force measurements were performed using EDL muscles as previously described(2). In brief, single EDL muscles

were surgically excised with ligatures at each tendon (5–0 silk suture) and mounted in a bath between a fixed post and force transducer (Aurora 300B-LR) operated in isometric mode. The muscle was maintained in modified Kreb's buffer solution (pH 7.2) containing (in mmol/l) 115 NaCl, 2.5 KCl, 1.8 $CaCl_2$, 2.15 Na_2HPO_4, 0.85 NaH_2PO_4, and maintained at 25°C under aeration with 95% O_2 – 5% CO_2 throughout the experiment. Resting tension and muscle length were iteratively adjusted for each muscle to obtain the optimal twitch force and a supramaximal stimulation current of 600mA was used for stimulation. After a five-minute equilibration, isometric tension was evaluated by 200 ms trains of pulses delivered at 10, 20, 40, 60, 80, 100, and 120 Hz. Length was determined with a digital microcaliper. After the experimental protocol, muscles were trimmed proximal to the suture connections, excess moisture was removed, and the muscle was weighed. The cross-sectional area for each muscle was determined by dividing the mass of the muscle (g) by the product of its length (L_0, mm) and the density of muscle (1.06 g cm^{-3}) and was expressed as millimeters squared (mm^2). Muscle output was then expressed as specific force (N/cm^2) determined by dividing the tension (N) by the muscle cross-sectional area(2).

Preparation of isolated skeletal muscle mitochondria. Skeletal muscle mitochondria were isolated from the plantar flexors (i.e. gastrocnemius, soleus, and plantaris) muscles of both control (right) and surgery (left) hindlimbs. To ensure sufficient mitochondrial yield was obtained, muscle was pooled from two animals for HLI studies. Upon dissection, muscle was washed in mitochondrial isolation medium (MIM) containing 300mM sucrose, 10mM HEPES, and 1mM EGTA. After washing, the muscle was minced on ice using fine tipped scissors for five minutes. The minced muscle

tissue was then trypsinized for two minutes before the addition of trypsin inhibitor. Trypsinized muscle was then washed and resuspended in MIM + bovine serum albumin (BSA, 1mg/ml) and homogenized on ice using a Teflon pestle and Wheaton overhead stirrer. The homogenate was centrifuged at 800xg to pellet non-mitochondrial myofibrillar proteins, nuclei, and other cellular components. The resulting supernatant was transferred to a pre-chilled oakridge tube and then centrifuged at 12,000xg to pellet mitochondria. Mitochondrial pellets were washed and gently resuspended in 5ml of MIM+BSA by pipetting. A second centrifugation was performed at 12,000xg, and the supernatant discarded. The final mitochondrial pellet was resuspended in 100μl of MIM and stored on ice until analysis (less than 1 hr). Mitochondrial protein content was determined by BCA protein assay (Pierce).

Mitochondrial respiration measurements. High-resolution O_2 consumption measurements[4] were conducted at 37˚C in buffer Z (in mmol/l) (105 K-MES, 30 KCl, 1 EGTA, mM K_2HPO_4, 5 $MgCl_26H_2O$, 0.5 mg/ml BSA, pH 7.1), supplemented with creatine monohydrate (20 mM), using the OROBOROS O2K Oxygraph. Mitochondrial respiration was measured in the presence of 0.5mM malate, 5mM pyruvate, 10mM glutamate, and 10mM succinate followed by the addition of 4mM ADP. Exogenous cytochrome c (1mM) was added to confirm intactness of mitochondrial membranes. Uncoupled respiration was assessed by the addition of 0.5μM FCCP. The rate of respiration was expressed as pmol/s/mg mitochondrial protein. Mitochondrial ATP:O ratio was assessed as previously described[5] with mitochondria incubated with 0.5mM malate, 5mM pyruvate, 10mM succinate, followed by the addition of 150μM ADP.

Blood lactate measurements. Mice were restrained at ~8:00am following a standard dark cycle with access to food. A ~2mm piece of the tail was clipped and blood collected into a glass capillary tube, immediately transferred to a microcentrifuge tube and allowed to clot for 30 min at room temp. Samples were centrifuged for 15 minutes at 3000xg, and serum was processed for blood lactate using a standard colorimetric assay kit (Sigma Cat # MAK064) according to the manufacturer instructions.

Immunofluorescence and histology. Skeletal muscle morphology and vessel density were assessed by standard light microscopy and IF microscopy. 10-µm-thick transverse sections from tibialis anterior (TA) frozen in liquid nitrogen cooled isopentane in optimum mounting medium (OCT) were cut using a Leica 3050S cryotome and collected on charged slides for staining. For morphological analyses, standard methods for hematoxylin and eosin (H&E) histological staining were performed and images were obtained at 4x and 20x magnification using an Evos FL Auto microscope (ThermoScientific).

Transmission Electron Microscopy. Anesthetized BL6J or BALB/c mice were intra-peritoneally perfused with PBS, followed by perfusion fixation with 4% PFA and 1.5% glutaraldehyde in 0.1 M Na-cacodylate buffer. The lower limb was removed and further fixed in the same solution at 4°C for 2 hours. Tibialis Anterior (TA) muscles were dissected and incubated overnight at 4°C in a secondary fix of 2.5% Glutaraldehyde in 0.1M Na-Cacodylate. Muscles were washed in Na-cacodylate buffer, post-fixed in 1% OsO_4 in 0.1 M Na-cacodylate for 1 hour, and rinsed in Na-cacodylate buffer. After dehydration with increasing concentrations of ethanol, muscles were embedded in Epon 812 resin. 65 nm ultrathin sections were stained with uranyl acetate and lead citrate and

visualized on the transmission electron microscope (FEI Technai Spirt BioTWIN, Hillsboro, OR, USA) at 120kV. All chemicals were purchased from Electron Microscopy Sciences, Hatfield, PA, USA.

Immunoblotting. Western Blotting was performed using standard methods. Frozen muscles or mitochondrial isolates were lysed in ice-cold RIPA buffer containing protease and phosphatase inhibitors. Protein concentrations were determined using a BCA protein assay (Pierce, ThermoFisher #23225). Proteins were then separated using an SDS-Page gel (Mini-Protean TGX, Bio-Rad #4561093). Blots were visualized with chemiluminescence using a Biorad Chemidoc imaging system. Densitometry was performed using ImageLab Software v5.2.1 (Biorad).

Primary muscle progenitor cell isolation and culture - Primary murine muscle precursor cells (mouse myoblasts) were derived from peripheral hindlimb muscles. Primary human muscle precursor cells (human myoblasts) were derived from fresh muscle biopsy samples. Briefly, peripheral skeletal muscle was dissected, trimmed of connective tissue, and placed in 10cm dishes containing ice cold sterile PBS. Organs were then transferred to separate 10cm dishes containing 5mL of pre-warmed MPC isolation medium (IM: DMEM with 4.5g/L glucose, supplemented with 1% Penicillin/Streptomycin/Amphotericin B) and any remaining connective tissue was trimmed. Organs were then transferred to a third 10cm dish containing 5mL of cold MPC IM, transported to the sterile culture hood, and minced for 2 minutes (per plate) using sterile razor blades. Minced slurry was transferred to 15mL tubes, 5mL additional MPC IM was added, tubes were inverted several times and centrifuged at 4°C for 3min at 700g to remove contaminants. MPC IM was subsequently aspirated and pellet was

resuspended in 10mL of MPC IM and inverted 5-10x to loosen pellet and mix before decanting into a 10cm culture dish. Tubes were subsequently rinsed in 8mL MPC IM to ensure all tissue was removed and 2mL of 1% pronase (Calbiochem #53702) was added to equal a final concentration of 0.1%. A sterile, low-profile magnetic stir bar was added and dishes were stirred at low rpm on a magnetic stir plate at 37°C and 5% CO2 for 1hr. Digested tissue slurry was then transferred to 50mL conical tubes and centrifuged for 4min at 800g at RT. Supernatant was aspirated and digest pellet was resuspended in 10mL MPC purification medium (PM: DMEM with 4.5g/L glucose, supplemented with 10% fetal calf serum and 1% Penicillin/Streptomycin/Amphotericin B). Suspension was then triturated approximately 20x through a blunt end pipetting needle attached to a sterile 30cc syringe. Suspension was then passed through a 100μM disposable Steriflip vacuum filter into a 50mL tube, including 3 successive 8mL washes of the sieve with pre-warmed PM, and subsequently centrifuged at RT 5min at 1000g. Cell pellet was then resuspended in 1mL FBS before addition to primary MPC growth medium (GM: Ham's F10, supplemented with 20% FBS and 1% Penicillin/Streptomycin/Amphotericin B, and supplemented immediately prior to use with 5ng/mL basic FGF). Cells were plated on collagen coated T150 flasks, allowed to adhere and proliferate for 3-days and subsequently trypsinized in 0.25% Trypsin/EDTA and pre-plated at 37°C and 5% CO2 for 1hr on an uncoated T150 flask to allow for fibroblast purification, supernatant containing MPCs was removed and centrifuged at 800g for 5min at RT prior to re-plating in MPC GM on collagen coated T150 flasks. After reaching approximately 70% confluence, MPCs were then rinsed 1x in sterile PBS and switched to differentiation medium for myotube formation (DM: DMEM with 4.5g/L

glucose, supplemented with 2% HoS and 1% Penicillin/Streptomycin/Amphotericin B and supplemented with 1% insulin/transferrin/selenium immediately prior to use). DM was changed every 24-hours.

Extracellular acidification rate assay. Primary MPC's isolated from all Polg genotypes were plated at 150,000 cells/well into Seahorse XF24 assay plates coated with entactin/collagen/laminin (EmdMillipore) and allowed to adhere overnight and differentiated by serum withdrawal for five days (DMEM + 2% horse serum). HUVEC's were seeded at 100,000 cells/well on 0.1% gelatin coated dishes. For AAV experiments, BALB/c primary muscle cells were seeded into XF24 assay plates at confluency, infected with AAV at MOI of 10,000, and differentiated via serum withdrawal (DMEM + 2% horse serum) for 5 days. Extracellular acidification rate, a measure of glycolytic flux, was assessed using a Seahorse XF24 machine. Measurements began in basal XF assay media (without glucose, pH = 7.4), followed by sequential additions of glucose (10mM), oligomycin (1μg/ml), and 2-deoxyglucose (100mM).

Hypoxia cell survival assay. Primary myoblasts were isolated from BALBc/J mice. Prior to seeding, cells were infected in T75 flasks with AAV-GFP or AAV-PFKFB3 at an MOI = 10,000. No virus control C2C12 cells were also used. Two days after AAV infection, C2C12 myoblasts were seeded at 10,000 cells/well in 96-well plates and allowed to adhere for 6 hours. Cells were rinsed with PBS provided fresh growth media (DMEM + 4.5g/L glucose, supplemented with 10% fetal bovine serum and 1% Penicillin/Streptomycin). Cells were then placed into a cakepan hypoxia chamber (StemCell Technologies) and flushed with nitrogen for 10 minutes. After 48hours in the chamber, cells were rinsed with PBS and fixed with ice-cold methanol/acetone (1:1).

Normoxic control plates were cultured under standard conditions. Fixed cells were stained with DAPI and 4x images were captured using automated routines on the Evos FL Auto microscope. Cell counts were determined using automated routine in Cell Profiler software (Broad Institute).

Statistics - Data are presented as mean ± SEM. Comparisons between 2 groups were performed by Student's 2-tailed *t*-test. Comparisons of data with more than 2 groups were performed using two-way ANOVA with Tukey's post-hoc multiple comparisons. Repeated-measures ANOVA was performed when appropriate. Nonparametric Mann-Whitney testing was used to determine differences between the distributions of necrosis scores between groups. In all cases, $P < 0.05$ was considered statistically significant.

References

1. Ryan TE, Yamaguchi DJ, Schmidt CA, Zeczycki TN, Shaikh SR, Brophy P, Green TD, Tarpey MD, Karnekar R, Goldberg EJ, Sparagna GC, Torres MJ, Annex BH, Neufer PD, Spangenburg EE and McClung JM. Extensive skeletal muscle cell mitochondriopathy distinguishes critical limb ischemia patients from claudicants. *JCI Insight*. 2018; Nov 2;3(21):e123235.
2. Ryan TE, Schmidt CA, Green TD, Spangenburg EE, Neufer PD and McClung JM. Targeted expression of Catalase to Mitochondria Protects Against Ischemic Myopathy in High Fat Fed Mice. *Diabetes*. 2016; Sep;65(9):2553-68.
